# Improved FBAM and GO/PO Method for EM Scattering Analyses of Ship Targets in a Marine Environment

**DOI:** 10.3390/s20174735

**Published:** 2020-08-21

**Authors:** Jinxing Li, Min Zhang, Wangqiang Jiang, Pengbo Wei

**Affiliations:** School of Physics and Optoelectronic Engineering, Xidian University, Xi’an 710071, China; jxli@xidian.edu.cn (J.L.); wqjiang@mail.xidian.edu.cn (W.J.); pbwei@xidian.edu.cn (P.W.)

**Keywords:** EM scattering, ship targets, marine environment, RCS

## Abstract

The combination of the fact-based asymptotic method (FBAM) and the geometrical optics and physical optics (GO/PO) hybrid method is an effective way to analyze the electromagnetic (EM) scattering from electrically large ship targets in a marine environment because it takes the multiple scattering of the ship targets into consideration as well as the coupling scattering field between the targets and the sea surface. However, regarding an electrically large marine scene that contains a large target, the occlusion judgement process for calculating the multiple scattering field and the coupling field makes it inefficient. To solve this problem, this paper proposes a physical mechanism-based improved method to reduce the invalid occlusion judgment between different patches on the composite ship–ocean scene, and this operation enhances the computational efficiency significantly. With the proposed method, radar cross section (RCS) results of different targets and composite ship–ocean scenes were calculated and compared with the original FBAM and GO/PO method. Numerical results showed that the proposed method had higher efficiency compared with the original method with the same good accuracy. In addition, synthetic aperture radar (SAR) images of a composite ship–ocean scene with different radar parameters and sea conditions were simulated with the proposed method for detection purpose. Finally, the proposed method was used to analyze the EM scattering characteristic of a marine environment with multiple ships.

## 1. Introduction

The study of microwave scattering from ship targets in a marine environment has been a challenge for a long time due to the complex structure of targets, the randomness of sea surfaces, and the interactions between them. In addition, the electrically large size of such a composite scene makes the scattering problem much more difficult. However, the related study is of great value in the field of target detection, target feature extraction, etc. Thus, this problem has been studied by many researchers and various of methods have been proposed to solve this problem despite its difficulty.

The existing methods can be roughly divided into three categories. The first include numerical methods such as the famous method of moments (MoM), the finite element method (FEM) [1,2], the finite-difference time-domain (FDTD) algorithm [3], the time domain integral equation (TDIE), and other methods that have been developed based on them such as the forward-backward method (FBM) [4], the multilevel fast multipole method (MLFMM) [5,6,7], etc. The second category is the combination of numerical methods and high-frequency approximation methods. For this kind of method, the scattering field from rough surfaces and the targets are always calculated with high-frequency approximation methods and numerical methods, respectively. In [8,9], the combination of the Kirchhoff approximation method (KAM) and the MoM or MLFMM were used to analyze the scattering from a target over a rough surface. Compared with the pure numerical methods, they show a higher efficiency. Even so, the combination methods suffer from huge computational burden when solving the scattering from electrically large ships in a marine environment at higher microwave bands. The third kind include the pure high-frequency approximation methods such as the four-path model (FPM) [10,11], the iterative physical optics (IPO) method [12], the ray-tracing based methods such as the shooting and bouncing ray (SBR) algorithm [13], and the geometrical optics and physical optics (GO/PO) method [14]. Among them, the GO/PO based methods are widely employed due to the lower memory costs, higher computation efficiency, intelligible mechanism, and reasonable results in practice. As for this kind of method, the total scattering field can be divided into three parts, namely, the direct scattering field from the sea surface, the direct scattering field from ship targets, and the coupling scattering field between them. The GO/PO method is used to predict the direct scattering field from the ship targets and the coupling scattering field. However, when calculating the higher-order scattering field from the ship targets and the coupling scattering field via the GO/PO method, the visibility judgement makes it inefficient if the size of the sea surface or the target is too large. Therefore, different methods are used to accelerate the GO/PO method such as the Kd-tree [15] and the Open Graphics Library (OpenGL) technology [16].

In this paper, another idea was proposed to accelerate the current GO/PO method by using the physical mechanism to reduce the invalid occlusion judgment between different patches, and this operation enhances the computational efficiency when dealing with the second-order scattering field from the ship target and the coupling scattering field between the ship target and the sea surface. Therefore, the proposed improved way was used to analyze the EM scattering from ship targets over a sea surface by combining with the fact-based asymptotic method (FBAM).

The rest of this paper is organized as follows. In Section 2, the original /PO method is introduced briefly, then the improved idea is described in detail. In Section 3, both the calculation accuracy and the efficiency of the improved method are compared with the original GO/PO method to show its good ability. Next, it is used to simulate synthetic aperture radar (SAR) images of a composite ship–ocean scene for detection purpose. In Section 4, the proposed method is used to analyze the EM scattering characteristic of a marine environment with multiple ships. Finally, this work is concluded in Section 5.

## 2. The Original and the Improved Methods

### 2.1. The Original FBAM and GO/PO Method for EM Scattering from a Composite Ship-Ocean Scene

When solving the scattering problems of an electrically large marine scene with ships using high-frequency methods, the total scattering field from a composite ship–ocean scene can be divided into three parts (i.e., the direct scattering field from the sea surface $E_{\mathrm{sea}}$, the scattering field from the ship target $E_{\mathrm{target}}$, and the coupling scattering field between the sea surface and the ship target). Regarding the combination of the FBAM and GO/PO hybrid method proposed in [14], the direct scattering field was calculated by the FBAM, and the scattering field from the ship target and the coupling field were estimated with the GO/PO method, which are briefly introduced in the following.

#### 2.1.1. FBAM for the Scattering Field from Sea Surface

The FBAM is a modified model based on the classical two scale model (TSM), which was first introduced in [17]. This method simplifies the real capillary waves only with their Bragg components according to the Bragg resonant hypothesis. Therefore, the whole sea surface can be generated with a larger sampling interval, which makes it able to analyze the scattering field from an electrically large scene efficiently. In addition, the scattering field (including the amplitude and phase) from a facet on the sea surface can be obtained. With FBAM, the scattering field from the *m*-th facet on the sea surface is

(1) $$E_{pp}^{m}=\frac{k^{2}(1-\varepsilon )\times \Delta x\Delta y\exp(jkR)}{j4Rn_{z}}\times \exp(-jq\times r_{0})F_{pp}\times I(\times )$$

where subscript pp=hh,vv indicates the polarization mode of the incident wave and the scattered wave, respectively; k is the incident wavenumber; ε is the relative dielectric constant of seawater; Δx,Δy are the sampling interval of the sea surface along the x and y directions, respectively; j is the imaginary unit; $n_{z}$ is the z-component of the normal vector; $q=k\left({\hat{k}}_{s}-{\hat{k}}_{i}\right)$ with ${\hat{k}}_{i},\;{\hat{k}}_{s}$ being the unit vectors of the incident and the scattered waves, respectively. $r_{0}$ is the wave vector of the sea facet, $F_{pp}$ is the polarization factor in global frame, and I(×) is related to the slopes and height of the sea facet. Both $F_{pp}$ and I(×) are available in [17]. Then, the total scattered field from the whole sea surface can be obtained by summing the scattered field from each facet:

(2) $$E_{pp}^{\mathrm{sea}}=\sum_{m=1}^{M}E_{pp}^{m}$$

where M is the total number of facets of the sea surface.

#### 2.1.2. Original GO/PO Method for Scattering Field from Ship Target and the Coupling Scattering Field

Compared with the PO method, the GO/PO hybrid method can estimate the first-order scattering field as well as the higher-order scattering field, thus it can be applied to analyze the scattering field from complex targets and the coupling field between a sea surface and a target on it.

As for the first-order scattering field from a target, the target should be divided into small triangle patches, and the visibility of each patch to the incident wave should be judged. Then, the first-order scattering field from a patch, which is induced by the incident wave, can be calculated by the PO method (i.e., [14]):

(3) $$E_{s}^{1}=j2k\psi_{0}\int_{S}{\hat{k}}_{s}\times [Z_{0}{\hat{k}}_{s}\times \left(\hat{n}\times H_{i}\right)]e^{-jkr\times \left({\hat{k}}_{i}-{\hat{k}}_{s}\right)}dS$$

where $\psi_{0}$ is the far-field Green function; $Z_{0}$ is the impedance of the free space; $\hat{n}$ is the unit normal vector of the facet; and r is its position vector. In numerical simulations, Equation (3) can be calculated with Gordon’s method [18].

Except for the first-order field, the multiple reflections make significant contributions to the scattering field for complex targets. To take this effect into consideration, the GO method was introduced to test the intersection of each reflection ray from a facet on the ship–ocean scene with another one. Taking the visibility of patch *n* to the reflected wave from patch *m* as an example, four conditions must be met so that the reflected wave from patch *m* can illuminate patch *n* [14,15]. First, the *m*-th patch must be illuminated by the incident wave, only in this case, it can produce a reflected wave and may generate induced current on patch *n*. In this premise, the direction of the unit reflected wave vector ${\hat{k}}_{r,m}$ must satisfy the condition ${\hat{k}}_{r,m}\times {\hat{n}}_{n}<0$ (${\hat{n}}_{n}$ is the normal vector of patch *n*), in other words, the reflected wave is likely to illuminate the outside surface of patch *n*. In addition, the reflected ray along the reflected wave vector direction must intersect with patch *n*. However, even if the reflected ray intersects with patch *n*, the reflected wave may be shaded by other patches. Therefore, another important condition is that it is not shaded by other patches when the reflected rays pass into the patch. Only the conditions above-mentioned were all met; patch *n* is illuminated by the reflected wave from patch *m*.

**Figure 1:**
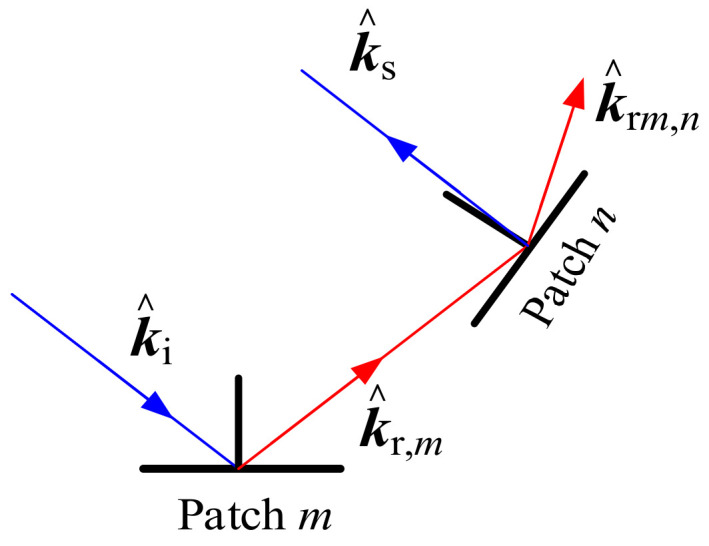
Illustration of the second-order scattering from patch *m* to patch *n*.

Figure 1 shows the second-order scattering field from patch *n* caused by the reflection wave from patch *m*. The reflected wave vector from patch *m* is ${\hat{k}}_{r,m}$, and it is reflected on patch *n* with a wave vector ${\hat{k}}_{r,mn}$. Then, the scattering field $E_{m,n}$ from patch *n* can be computed by the PO method. The second-order field from the target is

(4) $$E_{s}^{2}=\sum_{m=1}^{N}\sum_{n=1\;(n\neq m)}^{N}E_{m,n}\times I_{m,n}$$

where N is the total number of patches on the target; $I_{m,n}=1$ means that the reflected wave ${\hat{k}}_{r,m}$ from patch *m* is able to illuminate patch n, otherwise $I_{m,n}=0$. Through considering the multiple scattering between different patches on the target, the GO/PO method can handily treat the scattering from targets with complex structures.

Then, regarding the coupling field, the GO/PO method is still applicable. The coupling field can be divided into two parts. First, when the incident wave illuminates a facet on the sea surface, its reflected wave may illuminate a facet on the ship target. Second, the reflected wave from the ship target may also illuminate the sea surface. The calculation method is the same with that for estimating the second-order field from the ship target, the only difference is that one must consider the dielectric constant of the sea surface. Then, the total coupling scattering field is

(5) $$E_{\mathrm{cou}}=\sum_{m=1}^{M}\sum_{n=1\;}^{N}\left(E_{m,n}^{\mathrm{cou}}\times I_{m,n}^{\mathrm{cou}}+E_{n,m}^{\mathrm{cou}}\times I_{n,m}^{\mathrm{cou}}\right)$$

where $E_{m,n}^{\mathrm{cou}}$ is the scattered field from the *n*-th facet on the ship, which is induced by the reflected wave from the *m*-th facet on the sea surface; $E_{n,m}^{\mathrm{cou}}$ is the scattered field from the *m*-th facet on the sea surface induced by the reflected wave from the *n*-th facet on the target; and $I_{m,n}^{\mathrm{cou}}$ indicates whether the reflected wave ${\hat{k}}_{r,m}$ from facet *m* is able to illuminate patch *n.* If so, $I_{m,n}^{\mathrm{cou}}=1$, otherwise, $I_{m,n}^{\mathrm{cou}}=0$. The detailed implement process is available in [8].

### 2.2. The Improved GO/PO Method

**Figure 2:**
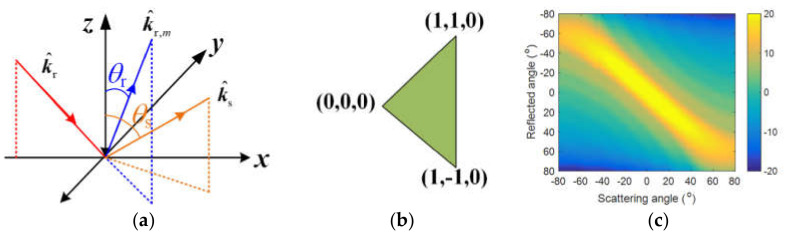
Geometrical configuration of the scattering problem (**a**), coordinates of the triangle patch (**b**), and the RCS results varying with the scattering angle and the reflection angle (**c**).

When calculating the second-order field from the ship target with the original GO/PO method, the visibility of each patch on the target to the reflection waves from other patches needed to be tested. Similarly, the visibility test should also be undertaken in the calculation process of the coupling scattering field between the ship target and the sea surface. However, if the angle between ${\hat{k}}_{r,mn}$ and ${\hat{k}}_{s}$ is too large, the second-order scattering field from patch *n*, which is induced by ${\hat{k}}_{r,m}$, is too weak and can be neglected. Here, ${\hat{k}}_{r,mn}$ is the second-order reflected wave vector induced by ${\hat{k}}_{r,m}$ on patch *n*. In order to prove this idea, the radar cross section (RCS) results of a small triangle patch (see Figure 2b) varied with scattering angle $\theta_{s}$ and reflection angle $\theta_{r}$, as shown in Figure 2c. In this simulation, the second order reflected wave on the small patch was ${\hat{k}}_{r,mn}=\left(\sin\theta_{r},0,\cos\theta_{r}\right)$, the normal vector of the triangle patch was ${\hat{n}}_{r}=\left(0,0,1\right)$, and the scattered vector was ${\hat{k}}_{s}=\left(\sin\theta_{s},0,\cos\theta_{s}\right)$. It was seen that the RCS results decreased with the increase in the difference between $\theta_{s}$ and $\theta_{r}$.

With this idea, the second-order field from the target and the coupling field can be described as

(6) $$E_{s}^{2}=\sum_{m=1}^{M}\sum_{n=1\;(n\neq m)}^{M}E_{m,n}\times I_{m,n}\times \chi \left(\alpha \right)$$

(7) $$E_{\mathrm{cou}}=\sum_{m=1}^{M}\sum_{n=1\;}^{N} [E_{m,n}^{\mathrm{cou}}\times I_{m,n}^{\mathrm{cou}}\times \chi \left(\alpha \right)+E_{n,m}^{\mathrm{cou}}\times I_{n,m}^{\mathrm{cou}}\times \chi \left(\alpha \right)]$$

where α is the angle between ${\hat{k}}_{r,mn}$ and ${\hat{k}}_{s}$, and

(8) $$\chi \left(\alpha \right)=\left\{\begin{array}{l} 1,\;\mathrm{if}\;\alpha <a_{0}\\ 0,\;\mathrm{else} \end{array}\right.$$

where $\alpha_{0}$ is a threshold angle and $\chi \left(\alpha \right)$ means that if the angle between ${\hat{k}}_{r,mn}$ and ${\hat{k}}_{s}$ is larger than $\alpha_{0}$, the contribution of ${\hat{k}}_{r,mn}$ to the second-order scattering field can be non-ignorable because it is too weak. Based on Figure 2c, we can see that the RCS results of the triangle patch shown in Figure 2b decreases with the difference $\Delta \theta =\left|\theta_{s}-\theta_{r}\right|$ between the scattering angle $\theta_{s}$ and reflection angle $\theta_{r}$. Generally, when Δθ=20°, the second-order scattering field from a patch, which is induced by ${\hat{k}}_{r,m}$, is too weak and can be neglected. Therefore, the threshold value can be set as 20°. However, if a higher accuracy is needed, the threshold value can be set as a larger value.

Based on Equations (6) and (7), if $\chi \left(\alpha \right)=0$, the visibility test no longer needs to be conducted. In this way, many invalid visibility tests can be neglected, and the computation efficiency can be enhanced. Finally, the RCS from a composite ship–ocean scene is

(9) $$\sigma =\underset{R\to \infty }{\lim}4\pi R^{2}\frac{{\left|E_{s}^{}\right|}^{2}}{{\left|E_{i}\right|}^{2}}=\underset{R\to \infty }{\lim}4\pi R^{2}\frac{{\left|E_{}^{\mathrm{sea}}+E_{s}^{1}+E_{s}^{2}+E_{\mathrm{cou}}\right|}^{2}}{{\left|E_{i}\right|}^{2}}$$

where $E_{i}$ is the incident electric field.

Finally, it should be noted that the proposed acceleration idea can also be further combined with the Kd-tree or the OpenGL technology. For example, when calculating the first-order scattering field, the visibility of each patch on the composite ship–ocean scene can be judged with the Kd-tree or OpenGL technology. However, this is beyond the purpose of this paper.

## 3. Validity and Application of the Improved Method

In this section, through different examples, the calculation accuracy of the improved GO/PO method for predicting the RCS from a pure target was proved first. Then, by combining with FBAM, the RCS results of composite ship–ocean scenes were calculated and compared with the original method to further prove the accuracy and efficiency of the proposed approach. Then, the proposed method was used to simulate the SAR images of a ship over a rough sea surface for the purpose of target detection.

(1) Example 1: A ship model

First, the radar cross section (RCS) of a simple ship model was calculated via the original GO/PO method, the improved GO/PO hybrid method, and the multi-level fast multipole method (MLFMM). The MLFMM is a fast algorithm based on the famous Method of Moments (MoM), which can reduce the storage requirements and low complexity of the matrix and vector multiplication effectively compared with MoM. As a low-frequency method, the good accuracy of MLFMM for solving electromagnetic scattering problems from objects has been proven in many studies [5,6,7]. Therefore, the results calculated by the MLFMM were regarded as a comparison.

**Figure 3:**
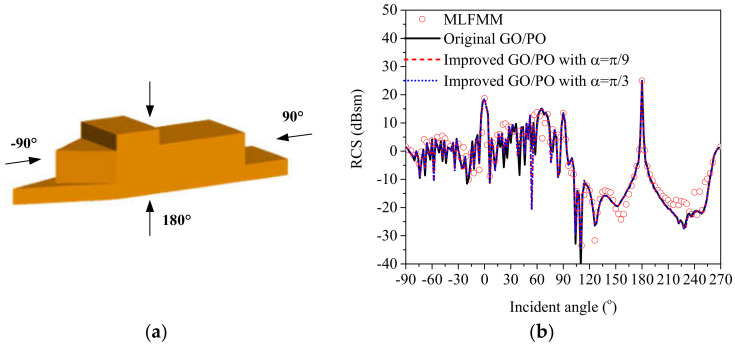
Geometrical structure of the ship model (**a**) and the RCS results vary with the incident angle (**b**).

The geometric dimensioning of the simple ship model is shown in Figure 3a, which was identical to that in Figure 2a in [19]. Figure 3b shows the monostatic VV-polarized RCS of the composite scene in the xoz plane at a frequency of 10 GHz, and the incident angle varied from −90° to 270° with an interval of 3°. The simulated results showed that the RCS results were largest near the normal incidence directions. The effectiveness of the improved GO/PO hybrid method was verified by good agreement with the MLFMM and the original GO/PO hybrid method. Moreover, the consumed time for calculating the RCS results with the MFLMM, the original GO/PO method, and the improved method are compared in Table 1 to show the good efficiency of the proposed method.

(2) Example 2: A ship target on a rough sea surface

**Figure 4:**
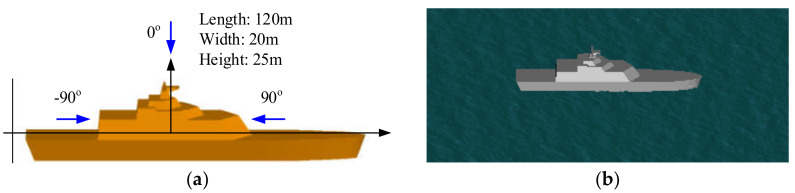
Geometry structure of the ship target 1 (**a**) and the composite scene (**b**).

**Figure 5:**
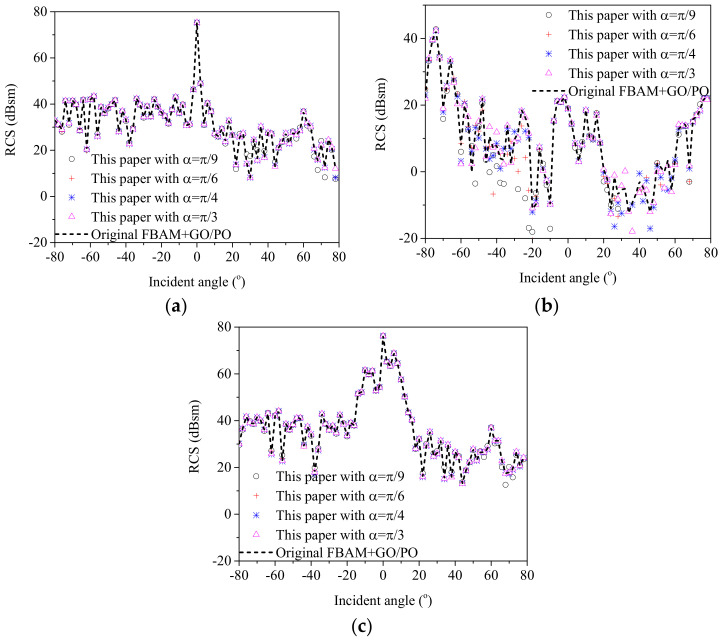
Comparison of calculation accuracy between the proposed way and the original GO/PO method. (**a**) The scattering filed from the ship target. (**b**) The coupling scattering field. (**c**) The total scattering field.

Figure 4 shows the geometry structure of a ship target on a sea surface, where the ship has a length of 120 m, a width of 20 m, and a height of 25 m. With the proposed method, the HH polarized backscattering RCS results corresponding to the scattering field from the ship, the coupling scattering field, and the total scattering field were calculated via the improved GO/PO method with different threshold angles. However, regarding electrically large objects (such as ships) at higher microwave bands, the MLFMM still suffers from a large computational burden. Therefore, for large ship targets, the good accuracy of proposed method can be proven only through a comparison with the original GO/PO method for large ships. The results were compared with those calculated by the original GO/PO method in Figure 5. In the simulation, the incident frequency was 10 GHz, and the incident angle varied from −80° to 80°. The results of the comparison showed the good ability of the proposed method to evaluate the scattering characteristics from a composite ship–ocean scene.

Furthermore, the computation time for calculating the above results with different methods are also compared in Table 2. It can be seen that the proposed way showed higher efficiency compared with the original GO/PO method. In addition, the calculation efficiency of the proposed method increases for a larger threshold value. However, if the threshold value is too large, the calculation accuracy decreases reversely. Therefore, an appropriate threshold value is necessary to balance the calculation accuracy and the calculation efficiency.

(3) Example 3: Another ship target on a rough sea surface

**Figure 6:**
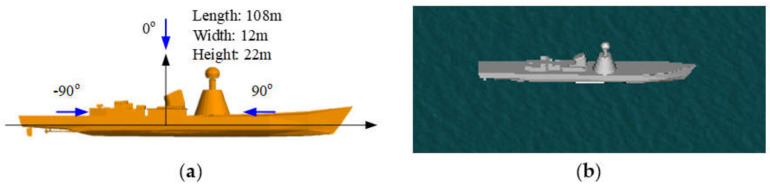
Geometry structure of the ship target 2 (**a**) and the composite scene (**b**).

**Figure 7:**
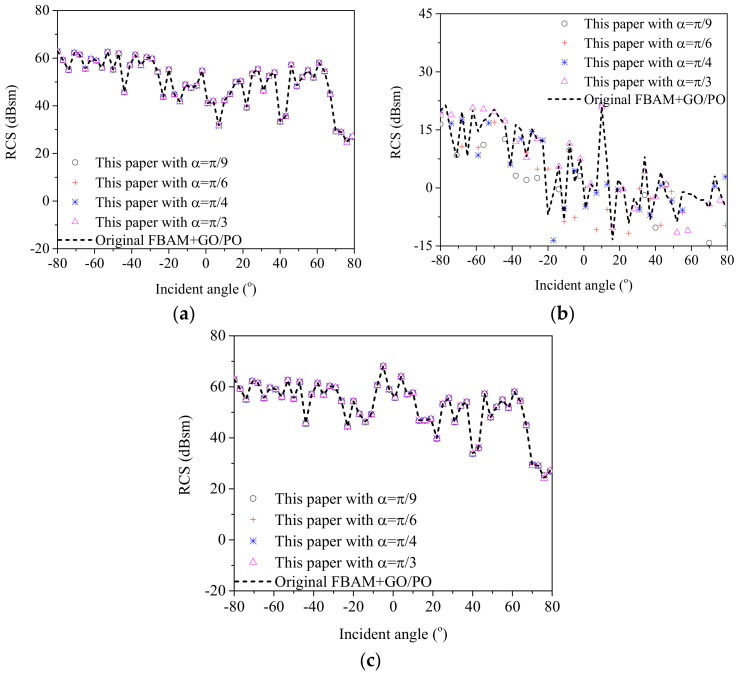
Comparison of calculation accuracy between the proposed way and the original GO/PO method. (**a**) The scattering filed from the ship target. (**b**) The coupling scattering field. (**c**) The total scattering field.

To show the good ability of the proposed method for analyzing the scattering from different shapes of targets, a composite ship–ocean scene with a different ship target is shown in Figure 6. The geometrical dimension of the ship target is illustrated in Figure 6a. With the proposed method, the HH polarized backscattering RCS results corresponding to the scattering field from the ship, the coupling scattering field, and the total scattering field were calculated via the improved GO/PO method with different threshold angles. The results were compared with those calculated by the original GO/PO method in Figure 7. The simulation parameters were the same as those in Figure 5. The comparison results showed that the proposed method performed well for the different ship targets.

(4) Application to SAR image simulation of a composite ship–ocean scene

**Figure 8:**
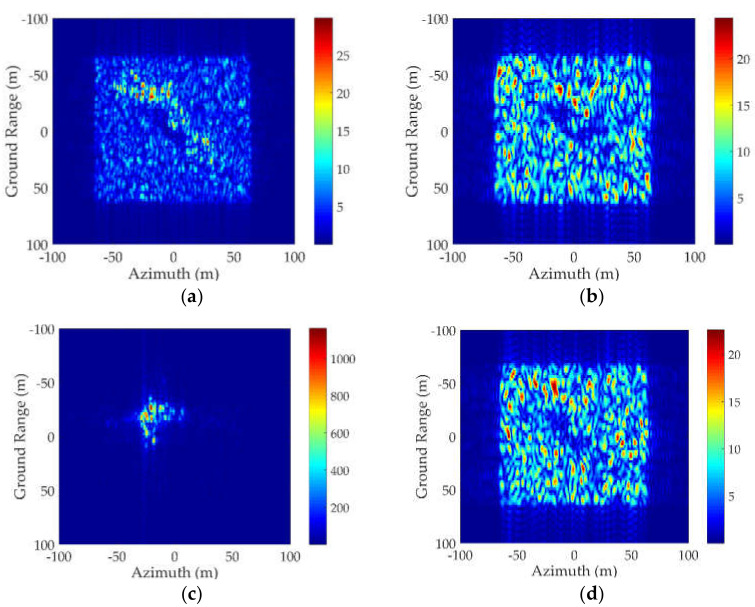
SAR images of composite ship–ocean scene with different parameters. (**a**) HH-pol, wind speed: 5 m/s, ship direction: 45° (**b**) VV-pol, wind speed: 5 m/s ship direction: 45° (**c**) HH-pol, wind speed: 5 m/s ship direction: 90° (**d**) HH-pol, wind speed: 10 m/s ship direction: 45°.

Nowadays, with the development of SAR, it has been applied to the detection of ship targets in a marine environment. Therefore, based on the proposed scattering model, SAR images of a composite ship–ocean scene under different radar parameters and sea states were simulated and are shown in Figure 8. The ship target is shown in Figure 4a, and the size of the sea surface was 128 m × 128 m, and the wind speeds were 5m/s for Figure 8a–c and 10 m/s for Figure 8d. The angles between the ship bow-stern direction and the flight direction of the SAR platform were 45° for Figure 8a,b,d and 90° for Figure 8c. The resolution was 2 m × 2 m. The central frequency of the SAR was 5 GHz and the incident angle was 40°.

First, by comparing the SAR images with different polarization modes, it was seen that with a HH polarized radar, the ship target was much easier to detect by a SAR, the reason being that the radar echo from the sea surface is much weaker than that for a VV polarized radar. Second, if the radar flies parallel to the ship target, the corner structures of the ship usually lead to a strong intensity, which makes it much easier to be detected by SAR images. However, under such a condition, the shape of the ship target in SAR images cannot reflects the whole image of the ship, making it difficult to recognize ship targets. Finally, by comparing Figure 8a,d, it can be seen that the wind speed has a great effect on SAR images because the echo from the sea surface is enhanced.

## 4. EM Scattering Characteristic of a Marine Scene with Multiple Ships

**Figure 9:**
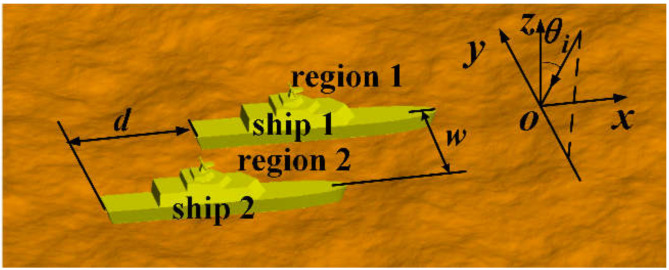
Composite marine scene containing two ships.

In this part, the improved method was used to analyze the EM scattering characteristics of a marine scene with multiple ships. Figure 9 shows the composite ship–ocean scene. The two ships were the same as those in Figure 4a. The center coordinates of ship 1 and ship 2 were (0, 0, 0) and (−d, w, 0), respectively.

**Figure 10:**
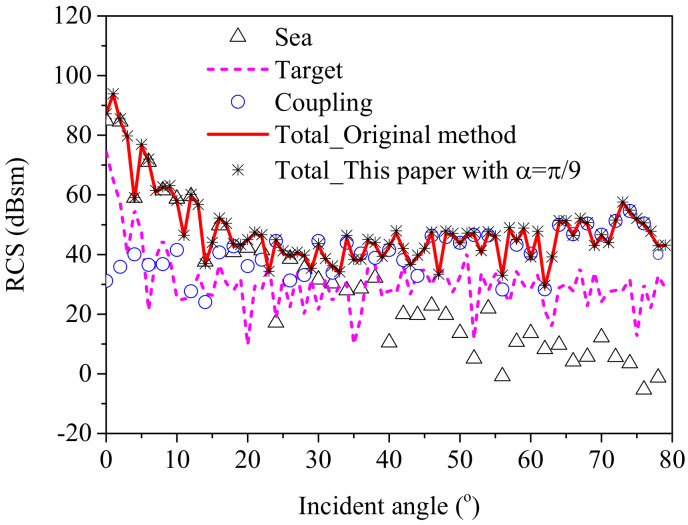
Comparison of different contributions in the ***yoz*** plane, *d* = 25 m, *w* = 30 m.

First, the RCS results from a composite ship–ocean scene (Figure 9 with *d* = 25 m, *w* = 30 m) was computed by the original method and the proposed method to show the good effectiveness of the proposed method for analyzing the scattering from multiple ships in a marine environment. Then, by comparing the three contributions to the total scattering field in Figure 10, it was observed that the direct scattering field from the sea surface and the ships reached the maximum value for a perpendicular incident angle. Furthermore, it can be concluded that at small incident angles, the scattering from the sea surface was dominant due to the large region of the sea surface. At larger incident angles, the coupling scattering field may exceed the scattering field from the targets, so the coupling scattering field is obviously non-ignorable especially for large incident angles.

**Figure 11:**
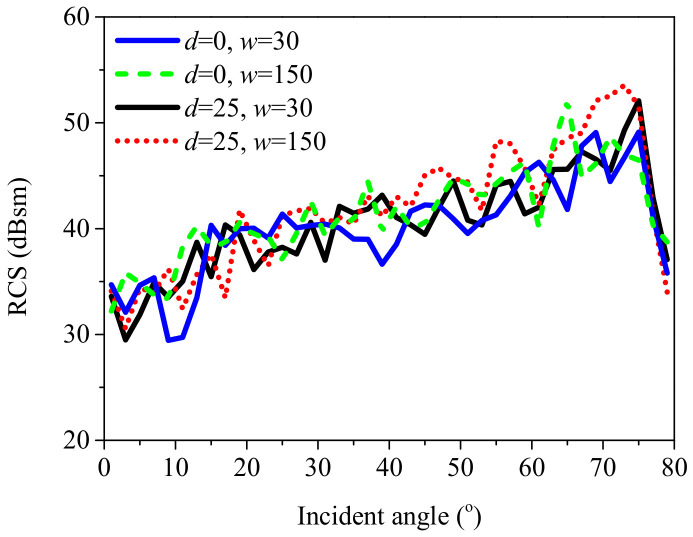
Comparison of the coupling scattering field in the ***yoz*** plane for different distances.

In addition, the coupling scattering field between the sea surface and the ships with different relative locations are compared in Figure 11. Due to the randomness of the sea surface, the RCS results from the coupling scattering are were averaged over 10 composite scene samples with different sea surface elevations. By comparing the coupling scattering field between the two ships for different intervals along the *y*-axis, one can see that there was no significant difference between the RCS results at small incident angles and very large incident angles. However, for intermediate incident angles, the coupling scattering field was larger for w = 150 m than that for w = 30 m. The reason is that when the incident wave illuminates the scene along the negative y-direction, the coupling scattering field contains two contributions: the coupling scattering field between ship 1 and the sea surface in region 1, and the coupling scattering field between ship 2 and the sea surface in region 2. If the distance between the two ships is large, the second contribution can be very large at intermediate incident angles. However, at small incident angles, it is weak because the reflected waves from the sea surface/ships illuminate the ships/sea surface only over a very small region. Furthermore, for large incident angles, the second contribution may vanish due to the shadowing effect.

**Figure 12:**
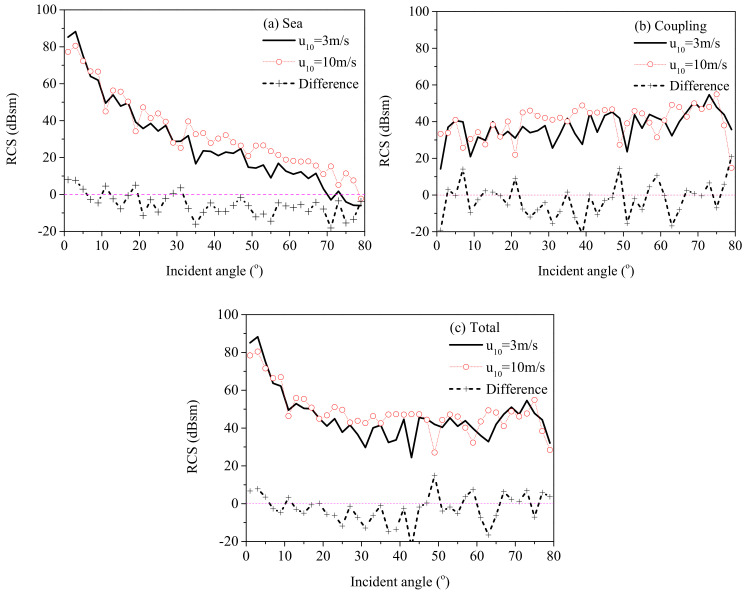
Comparison of RCS results in the ***yoz*** plane under different wind speeds. *d* = 25 m, *w* = 30 m. (**a**) RCS of the sea surface. (**b**) RCS from the coupling field. (**c**) RCS from the total field.

Furthermore, the RCS from the sea surface, the coupling field, and the total field under wind speeds of 3 m/s and 10 m/s are compared in Figure 12 to show the influence of the sea state. The difference $\Delta {=\mathrm{RCS}(u}_{10}=3)-{\mathrm{RCS}(u}_{10}=10)$ between the RCS results under different wind speeds is also shown in Figure 12. In Figure 12a, the RCS of the sea surfaces decreased for a higher wind speed in the specular directions, but increased at other incident angles, which is a logical phenomenon that in specular directions, the specular reflection dominates, thus the scattering field from a calm sea surface is stronger. However, for large incident angles, the diffuse scattering is the main mechanism. Therefore, the RCS increases with the wind speed. For the coupling scattering field, it was observed that the RCS from a rougher sea surface was higher than that of a calm sea surface at intermediate incident angles. Nevertheless, the difference was not obvious at small or large incident angles. Regarding the total scattering field in the ***yoz*** plane, Figure 12 shows that the scattering field from the sea surface and the coupling scattering field are the main contributions to the total scattering field at small and large incident angles, respectively. Thus, it has a similar trend with the scattering field from the sea surface at small incident angles, whereas it has the same trend with the coupling scattering field at intermediate and large incident angles.

## 5. Conclusions

In this paper, an improved way based on GO/PO method was proposed to investigate the scattering field from complex targets and the composite scenes. With the proposed approach, RCS results from a ship model and a composite ship–ocean scene were computed and compared with those calculated by the original GO/PO method. Numerical results showed that the proposed method showed good accuracy and the calculation efficiency increased when compared with the original GO/PO method because many invalid visibility tests can be avoided. The proposed can be further accelerated with GPU and the graphical occlusion technique. Finally, the proposed method was applied to analyze the scattering field from a composite scene containing two ships. The simulated results showed that the coupling scattering field between different ships was weak in the backscattering configuration, especially for a larger distance between them and a smaller incident angle. Importantly, the coupling scattering field between the sea surface and the ship targets was non-ignorable, especially for large incident angles. All the numerical results showed the validity of the proposed model.

## Figures and Tables

**Table 1:** Comparison of calculation efficiency between the MFLMM, the original GO/PO method, and the proposed method.

MLFMM	Original Method	This Paper with Different $\alpha_{0}$	
		π/9	π/3
51.57 h	0.195 s	0.137 s	0.159 s

**Table 2:** Comparison of the calculation efficiency between the proposed way and the original GO/PO method.

	Computation Time (s)				
	Original Method	This Paper with Different $\alpha_{0}$			
		π/9	π/6	π/4	π/3
First-order field from target	67.248	63.462	70.42	69.86	68.985
Second-order field from target	192.05	76.699	99.20	118.81	142.309
Coupling field	11,983.1	5730.88	7363.49	8268.11	9632.31
